# Significant Associations of SOX9 Gene Polymorphism and Gene Expression with the Risk of Osteonecrosis of the Femoral Head in a Han Population in Northern China

**DOI:** 10.1155/2016/5695317

**Published:** 2016-12-07

**Authors:** Yang Song, Zhenwu Du, Ming Ren, Qiwei Yang, Yujie Sui, Qingyu Wang, Ao Wang, Haiyue Zhao, Jincheng Wang, Guizhen Zhang

**Affiliations:** ^1^Department of Orthopedics, Second Clinical College, Jilin University, Ziqiang Street 218, Changchun 130041, China; ^2^Engineering Research Center of Molecular Diagnosis and Cellular Treatment for Metabolic Bone Diseases in Jilin Province, Ziqiang Street 218, Changchun 130041, China; ^3^Research Center, Second Clinical College, Jilin University, Ziqiang Street 218, Changchun 130041, China

## Abstract

Sex determining region Y-box 9 (SOX9) is a key transcription factor involved in cartilage formation during the embryonic development stage and cartilage growth and repair after birth. To explore the roles of polymorphism and expression of the SOX9 gene in the development of osteonecrosis of the femoral head (ONFH), we analyzed the polymorphism of rs12601701 [A/G] and rs1042667 [A/C] and the serum protein expression of the SOX9 gene in 182 patients with ONFH and 179 healthy control subjects. Results revealed that the A-A haplotype of SOX9 gene as well as the GG and AA genotypes of rs12601701 was significantly associated with increased ONFH risk (*P* = 0.038) and the risk of bilateral hip lesions of ONFH (*P* = 0.009), respectively. The C-A, A-A, and A-G haplotypes were also statistically associated with the decreased and increased risk of bilateral hip lesions of ONFH (*P* = 0.03, *P* = 0.048, and *P* = 0.013), respectively, while the A-A haplotype closely related to the clinical stages of ONFH (*P* = 0.041). More importantly, the serum SOX9 protein expression of the ONFH group was greatly decreased compared to control group (*P* = 0.0001). Our results first showed that the gene polymorphism and gene expression of SOX9 were significantly associated with the risk and clinical phenotypes of ONFH and also indicate that the SOX9 gene may play a key role in the development of ONFH.

## 1. Introduction

ONFH is a disorder caused by multiple factors and pathological processes. Both complex genetic and environmental factors have been implicated to associate with the risk of ONFH [1, 2]. A series of gene polymorphism, plasminogen activation/inhibition, angiogenesis, lipid metabolism, type II collagen, cytokines, growth factors, and so forth has been gradually proposed to be correlated with ONFH risk [3–7]. However, the previous researches were focused on SNPs genotypes and few results involved in the gene expression, gene function, and the clinical phenotypes of ONFH. Thus, these results could not well explain the roles of the genes polymorphism in the development of ONFH.

The main pathological changes of ONFH are related to lesions in the articular cartilage during both the ischemic necrosis and repair stages, in particular, the roles of the subchondral bone lesion during the development of ONFH [8, 9]. SOX9 is a key transcription factor in cartilage formation during the embryonic development stage as well as a key gene in cartilage growth after birth. SOX9 also plays important roles in cartilage and bone metabolism [10]. Cartilage and bone formation during endochondral ossification is a gradual process of cell differentiation. The differentiation of bone marrow mesenchymal stem cells (BMSCs) into chondrocytes is regulated by multiple factors and signaling pathways, including transcription factor SOX9 and Runx2. However, the association of SOX9 gene polymorphism with ONFH risk has not been reported. In view of the critical roles of SOX9 in cartilage and bone metabolism, two tag SNPs, rs12601701 [A/G] in the promoter and rs1042667 [A/C] in the 3′ UTR region of the SOX9 gene, were selected to investigate the gene polymorphism and gene expression and their associations with the clinical phenotypes of ONFH.

## 2. Materials and Methods

### 2.1. Participants

A total of 182 unrelated patients with ONFH (126 men, 56 women; age: 53.50 ± 12.63 yr) were consecutively enrolled at the Department of Orthopedics at the Second Clinical College of Jilin University (Changchun, China) from March, 2014 to June, 2015. Patients with ONFH that was caused by direct trauma and patients with ONFH concurrent with cardiovascular diseases, congenital diseases, HIV infection, diabetes mellitus, renal dysfunction, or cancer were excluded. The diagnosis of ONFH was established by evidence of osteonecrosis using plain radiographs in stages 2, 3, and 4 of the Ficat Classification system [11]. On the basis of the patient's detailed medical history and etiological factors, ONFH cases were classified into one of the following subgroups: alcohol-induced (68 cases, 38.42%), idiopathic (67 cases, 37.85%), and steroid-induced osteonecrosis (45 cases, 25.42%). Steroid-induced osteonecrosis was defined by a history of taking prednisolone cumulative 2000 mg or an equivalent over 21 days. Alcohol-induced osteonecrosis was defined by the consumption of more than 900 mL of pure ethanol per week. The course of ONFH ranged from 0.5 months to 240 months (mean 72.45 months), and the clinical stages of ONFH consisted of 13 cases of stage II (7.34%), 52 cases of stage III (29.38%), and 112 cases (63.28%) of stage IV. There were 6 cases of ONFH patients who failed to undergo the clinical stages or aetiological classification because of defect plain radiographs or unclear aetiological factors.

Moreover, a total of 179 unrelated health control subjects (114 men, 65 women; age: 52.58 ± 11.08 yr) who were age- and sex-matched with the ONFH group were consecutively enrolled at the Health Examination Center of the Second Clinical College of Jilin University (Changchun, China) from October 2014 to December 2014. Health control subjects were defined in the following manner: they had no hip pain, their fasting blood glucose, triglyceride and total cholesterol levels in serum were within normal reference value range, their abdominal ultrasound examination and chest X-ray radiography were normal, and they did not suffered from chronic diseases, such as cardiocerebral vascular diseases and cancers. All participants were Han Chinese from northeast China. The study was approved by the ethics committee of the Second Clinical College of Jilin University, Changchun, China, and conformed to the current ethical principles of the Declaration of Helsinki. All participants provided informed consent for their participation in the study.

### 2.2. Genomic DNA Extraction and SNP Selection

Approximately 2 mL of venous blood was collected from all of the participants after a minimum of 10 h fasting. Genomic DNA was extracted from whole blood samples using a genomic DNA extraction kit (DP318, TianGen, Beijing, China) following the manufacturer's protocols. The HapMap database and related literature were used to select the tag SNPs in the SOX9 gene; the population distributions of the tag SNPs in different countries, nationalities, and regions, especially in Asian populations, were also analyzed. The two tag SNPs in the SOX9 gene, rs12601701 [A/G] in 3′UTR region and rs1042667 [A/C] in promoter region, were chosen based on Linkage Disequilibrium (LD) analysis by HapMap (http://www.hapmap.org/index.html.en). The selection criteria of tag SNPs included *r* ^2^ > 0.8 or *D*′ = 1; minor allele frequencies > 0.05; rs12601701 [A/G] and rs1042667 [A/C] were from different Haplotype Blocks of SOX9 gene; minor allele G frequencies of rs12601701 and minor allele A frequencies of rs1042667 are 46% and 48%, respectively, in Chinese population.

### 2.3. Genotyping

The detection primer, probe sequence, and product size after ligase reaction of the rs12601701 [A/G] and rs1042667 [A/C] SNPs are shown in Table 1. PCR reactions were performed in a buffer containing 1 *μ*L of DNA, 1.5 *μ*L of MgCl_2_, 0.3 *μ*L of dNTPs, 0.15 *μ*L of primer mix, and 0.3 *μ*L of Taq DNA ligase in a final reaction volume of 15 *μ*L (all reagents from ABI, CT, USA). The reaction mixture was heated to 94°C for three minutes for denaturation and then subjected to 35 cycles of 94°C for 15 seconds, annealing at 54°C for 15 seconds, and extension at 72°C for 30 seconds, followed by a final extension step at 72°C for 5 min. The specific amplified fragments were used in an LDR assay to identify the mutations associated with the rs12601701 and rs1042667 SNPs. The LDR assay was performed in a reaction volume of 10 *μ*L that contained 3 *μ*L of PCR product, 1 *μ*L of 10x ligase reaction buffer, 0.125 *μ*L of (40 U/*μ*L) Taq DNA ligase, and 0.01 *μ*L of probe (10 pmol)/each probe; deionized H_2_O was added to a final volume of 10 *μ*L. The ligation reaction was performed using a GeneAmp PCR System 9700 (ABI Company, CT, USA) with the following temperature program: 2 min at 95°C and 30 cycles of 30 s at 94°C and 33 min at 56°C. The products were analyzed using an ABI PRISM 3730xl DNA sequencer (ABI Company, CT, USA). CHROMAS software was used to analyze the sequencing peak chart.

### 2.4. Serum SOX9 Protein Expression

Serum SOX9 protein expression was detected using ELISA Kits (DRE10118, Solarbio Company, Shanghai, China) according to the manufacturer's protocols. Briefly, the assay range of SOX9 was 60 ng/L~3600 ng/L. Set standards wells on the ELISA plates coated (density: 3600 ng/L, 2400 ng/L, 1200 ng/L, 600 ng/L, and 300 ng/L). Set blank wells separately (blank wells do not add sample and HRP-conjugate reagent; another step is the same.). Test sample well, add sample dilution 40 *μ*L to sample wells, and then add testing sample 10 *μ*L (sample final dilution is 5-fold). Incubate for 30 min at 37°C, wash with washing buffer, repeat 5 times, add HRP-conjugate reagent 50 *μ*L, incubate for 30 min at 37°C, wash 5 times, add Chromogen Solutions A and B 50 *μ*L separately, evade the light preservation for 15 min at 37°C, and add Stop Solution 50 *μ*L to each well. After adding Stop Solution within 15 min, take the blank well as zero and read absorbance at 450 nm using microplate reader of multiwavelength (Varioskan, Flash, Thermo Scientific, Waltham, USA).

### 2.5. Statistical Analysis

Shesis software (http://analysis.bio-x.cn/SHEsisMain.htm) was used to analyze the Hardy-Weinberg equilibrium and haplotypes between the ONFH and control groups. Logistical regression analyses were used to calculate the odds ratios (OR), 95% confidence intervals (CI), and corresponding *P* values of each SNP, controlling for age and sex as covariates. The genetic models of dominant, recessive, and codominant were considered, and the genotypes were given codes of 0, 1, and 2; 0, 1, and 1; or 0, 0, and 1 in the codominant, dominant, and recessive models, respectively. SPSS10.0 software was used to analyze the serum SOX9 protein expression between the ONFH and control groups and the associations of protein expression and genotypes with clinical phenotypes of ONFH, using the Student's *t*-test, ANOVA, and *χ* ^2^ test, respectively. Shesis software (haplotype analysis) was also used to analyze the association of the haplotypes with the clinical phenotypes. A *P* value of <0.05 was considered statistically significant.

## 3. Results

### 3.1. The Genotypes and Allele Frequencies of rs12601701 and rs1042667 of SOX9 Gene

SNP IDs, locations, HWE, and the results of the logistical regression analyses of the genotyped SNPs are presented in Table 2. The genotypes and allele frequencies of the SOX9 SNPs between ONFH and control groups were not statistically significant. However, in the ONFH idiopathic subgroup, the allele frequency and the recessive model of rs1042667 were significantly associated with decreased and increased risk of ONFH, respectively; OR (95% CI): 0.656 (0.437~0.982) and *P* = 0.040; 1.376 (1.005~1.883) and *P* = 0.047, as shown in Table 3.

### 3.2. The Haplotypes of rs12601701 and rs1042667 of SOX9 Gene

There are four haplotypes, A-A, A-G, C-A, and C-G, between rs1042667 and rs12601701. The frequency of the A-A haplotype in the ONFH group (5.9%) was significantly higher than that of the control group (2.8%, *P* = 0.038), as shown in Table 4.

### 3.3. The Associations of the Genotypes and Haplotypes of rs12601701 and rs1042667 with the Clinical Phenotypes of ONFH

Correlation analysis of the SOX9 genotypes with the course, etiological classification, unilateral or bilateral hips lesions, and clinical stages of ONFH revealed that, in the rs12601701 GG and AA genotype carriers, the frequency of bilateral hip lesions was significantly higher and lower than that of unilateral hip lesions, respectively (*P* = 0.009), as shown in Table 5. Correlation analysis between the SOX9 gene haplotypes and the clinical phenotypes of ONFH also showed that, in the patients carrying the A-A or C-A haplotype, the frequency of bilateral hip lesions was statistically decreased compared with the frequency of unilateral hip lesions (*P* = 0.032 and *P* = 0.048, resp.), while in the A-G haplotype carriers, the frequency of bilateral hip lesions was significantly increased compared to that of unilateral hip lesions (*P* = 0.013; Table 6). In the patients with the A-A haplotype, the frequency of stage IV lesions was statistically lower than that of stage III lesions (*P* = 0.041; Table 6).

### 3.4. Serum SOX9 Protein Expression and Its Correlation with Gene Polymorphism and the Clinical Phenotypes of ONFH

**Figure 1:**
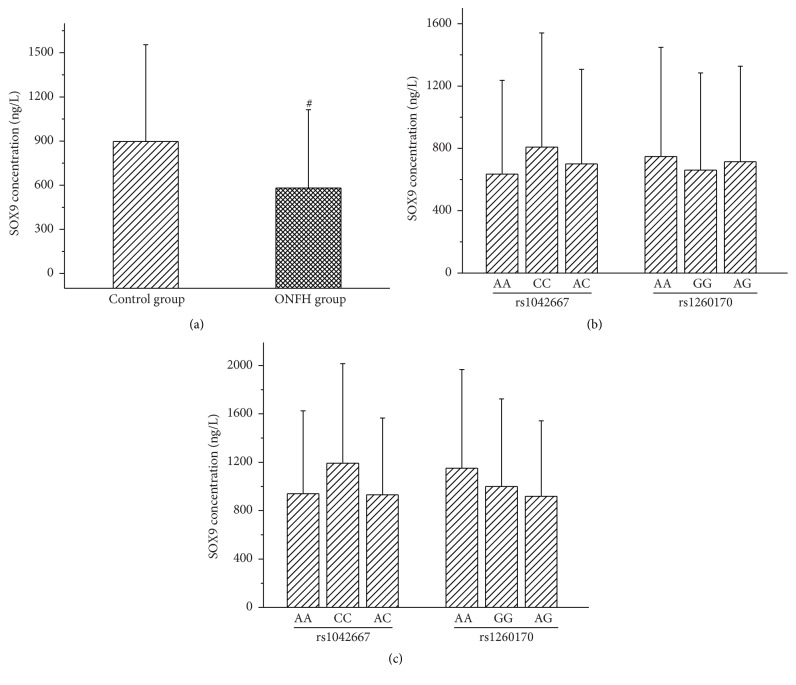
Serum protein expression of the SOX9 gene and the association of protein expression with SOX9 genotypes of ONFH. (a) The serum protein expression of the SOX9 gene between ONFH patients and control groups. ^#^ *P* = 0.0001, compared with control group. (b) Association of the SOX9 genotypes with serum SOX9 protein expression of the ONFH group. (c) Association of the SOX9 genotypes with serum SOX9 protein expression of the control group. The error bars in figures represent standard deviation.

**Figure 2:**
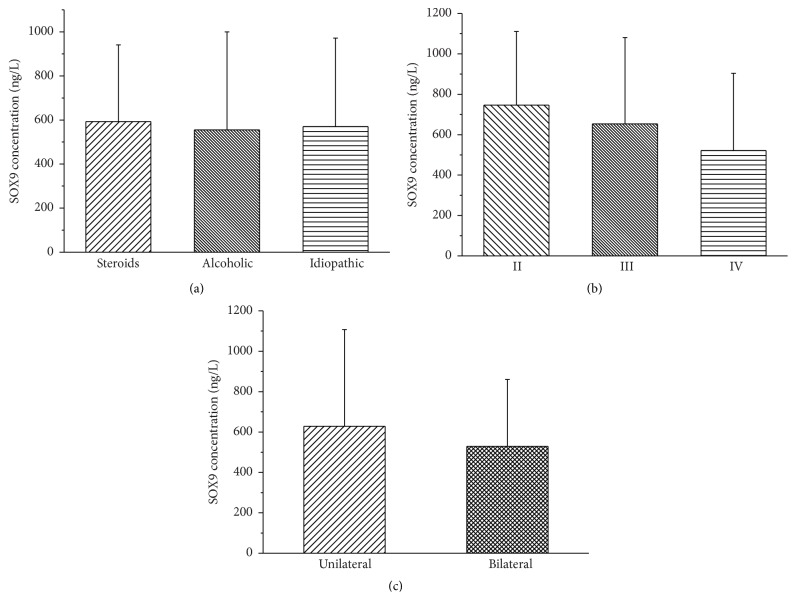
Association of SOX9 protein expression with the clinical phenotypes of ONFH. (a) Association of SOX9 protein expression with the clinical etiological classification of ONFH. (b) Association of SOX9 protein expression with the clinical stages of ONFH. (c) Association of SOX9 protein expression with unilateral hip or bilateral hips lesions in ONFH patients. The error bars in figures represent standard deviation.

The serum protein expression of SOX9 gene in the ONFH group was significantly lower than that of the control group (*P* = 0.0001). The expression of SOX9 protein in the different genotypes was not statistically significant (Figure 1). Although the correlation analyses between SOX9 protein expression and the etiological classification, clinical stage, and bilateral or unilateral hip lesions of ONFH failed to show statistical significance the serum SOX9 protein levels revealed a gradually decreased tendency from stage II to stage IV or from the subgroup of bilateral hip lesions to unilateral lesions subgroup (Figure 2).

## 4. Discussion

The SOX9 gene located in chromosome 17q24.3 is a critical gene related to early embryonic development. SOX9 participates in various developmental processes, including sex determination and cartilage formation. The upstream sequence mutation of the SOX9 gene causes abnormal cartilage formation (campomelic dysplasia, CD) [12, 13]. The SOX9 gene is expressed in multiple adult tissues and in the brain, liver, kidney, long bones, and cartilage in the stationary and proliferation phases of the fetus [14]. SOX9 binds to the specific cartilage cell enhancement factor, collagen type II alpha1 (COL2*α*1), and the promoter of the parathyroid hormone-related protein (PTHrP) gene and upregulates their expression and activities [15, 16]. The activation of Wnt signaling pathway by SOX9 plays important roles in the regeneration of cartilage cells, joint formation, and fracture repair [17–19]. As a member of the transforming growth factor-(TGF-) *β* family and a key regulator of fracture repair, bone morphogenetic protein- (BMP-) 2 significantly enhances the expression of the SOX9, type II collagen, and proteoglycan genes in intervertebral disc cells [20].

In consideration of the key roles of SOX9 in regulating cartilage and bone metabolism, our investigation selected two tag SNPs of SOX9 gene, rs1042667 (A/C) in the 3′UTR region and rs12601701 (A/G) in the promoter region, and analyzed their association of the genotypes, allele frequencies, and haplotypes with ONFH risk in 182 patients and 179 control subjects. The results showed that the frequency of the A-A haplotype in the ONFH group was significantly higher than that of the control group, indicating that the A-A haplotype is a risk haplotype of ONFH and the carriers with the A-A haplotype may associate with the increased ONFH risk. To further evaluate the correlation between SOX9 gene polymorphism and the development of ONFH, we also analyzed the association of the genotypes and haplotypes of the SOX9 gene with the clinical phenotypes of ONFH. The results revealed that in the GG and AA genotype carriers of rs12601701 the frequency of bilateral hip lesions was significantly increased and decreased, respectively, compared to that of unilateral hip lesions, suggesting that the ONFH patients carrying the rs12601701 GG or AA genotype have an increased or decreased genetic tendency toward bilateral hip lesions, respectively. Thus, we confirmed the significant association of the SOX9 genotypes with the hip lesions of ONFH.

In addition, the haplotypes of the SOX9 gene also correlated with the clinical phenotypes of ONFH. In the patients carrying the A-A or C-A haplotype, the frequency of bilateral hip lesions was obviously decreased compared with that of unilateral hip lesions, whereas, in the patients with the A-G haplotype, the frequency of bilateral hip lesions was statistically increased compared with that of unilateral hip lesions. Furthermore, in the ONFH patients with the A-A haplotype, the proportion of stage IV hip lesions was also significantly lower than that of stage III hip lesions. These results first suggest that the A-A and C-A haplotypes may be protective haplotypes against hip lesion progression, while the A-G haplotype may be a risk haplotype of hip lesion progression.

To explore the effects of SOX9 gene polymorphism on gene expression, we further detected SOX9 protein expression in serum. The result showed that serum SOX9 protein expression in the ONFH group was significantly decreased compared to the control group (*P* = 0.0001). Although there was a larger SD due to individual differences, SOX9 protein expression showed that the statistical significance as well as the serum level of SOX9 protein of ONFH group was decreased to 61% of the control group, which first confirmed that the serum SOX9 protein level in the patients with ONFH was greatly reduced. A study of SOX9 protein expression in both surgically resected tumor tissue and serum specimens of hereditary multiple myeloma (HME) confirmed the increased SOX9 expression levels in the HME sera and tissues, compared with those of the control group. Moreover, the expression of SOX9 protein in the serum was positively correlated with its expression in the tissue, indicating that the expression of SOX9 was involved in the pathogenesis of HME [21].

More significantly, our results not only showed that the genotypes and haplotypes of the SOX9 gene were closely related to the risk and clinical phenotypes of ONFH but also revealed that the abnormal decrease of serum SOX9 protein expression is associated with the development of ONFH. As an important consideration of this study, the mutation of the tag SNPs in the 3′UTR and promoter regions of the SOX9 gene may largely affect its gene expression efficiency and gene function. Consistent with the expected results, we confirmed that the expression of SOX9 protein was remarkably decreased in ONFH patients. Furthermore, in spite of no statistical significance, the serum SOX9 protein levels were a gradually lower tendency from stage II to stage IV of ONFH as well as from the subgroup of bilateral hip lesions to the subgroup of unilateral lesions. These results suggest that the decreased expression of the SOX9 gene may play the critical roles in the bone and cartilage lesions during the development of ONFH.

In the thickened cartilage layer of an experimental ONFH pig model, the expressions of the SOX9 and hypoxia inducible factor-1*α* (HIF-1*α*) genes were significantly upregulated and the target genes expressions regulated by SOX9, type II collagen, and aggrecan gene were also increased, which suggested that HIF-1*α*-mediated SOX9 gene expression enhanced the transcriptional activity of SOX9, resulting in a protective effect on the cartilage of ONFH [22]. A similar study in vitro also showed that BMSCs cultured for 14 days in hypoxic conditions (3% O_2_) developed significantly more cell colonies than that of BMSCs cultured under normal oxygen conditions (21% O_2_). Furthermore, after three weeks of inducing cartilage culture, the cartilage formation ability of BMSCs cultured in hypoxic conditions was also strongly increased compared with that of BMSCs cultured under normal oxygen conditions, with the obvious increase in the mRNA expression of SOX9, HIF-1*α*, type II collagen, and proteoglycan. These results also indicated the possible role of SOX9 in promoting the repair of articular cartilage during the development of ONFH [23].

The molecular mechanism of campomelic dysplasia/autosomal sex reversal caused by SOX9 gene mutation shows that two heterozygous mutations, F154L and A158T, destroy the alpha helix of the HMG domain of SOX9. In cultured cells, exact nuclear localization was observed for wild type SOX9 and the F154L mutant. However, the A158T mutant showed a 2-fold reduction in nuclear import efficiency, DNA binding was also drastically reduced in both mutants, but transcriptional activation in cultured cells was only reduced to 26% of wild type activity in F154L and to 62% in A158T, suggesting that a small loss of SOX9 transactivation activity could sufficiently disrupt the proper regulation of target genes during bone formation [24]. Unlike how CD is caused by SOX9 gene mutations, ONFH is a complex disorder caused by genetic factors and environmental factors; that is, the development of ONFH is associated with multiple microeffective genes and environmental factors. Further exploration of the interactions of SOX9 with other genes and environmental factors will be useful to elaborate the roles of SOX9 gene in the molecular pathogenesis of ONFH.

## 5. Conclusions

In conclusion, we first reported the association of SOX9 gene polymorphism and gene expression with the risk and development of ONFH. Our results demonstrated that the polymorphism of rs1042667 and rs12601701 in the SOX9 gene is significantly associated with the development of ONFH. Moreover, we confirmed that the serum SOX9 protein expression of ONFH patients was greatly reduced and was closely correlated with the increased risk of ONFH. These results suggest that the SOX9 gene may play a key role in the development of ONFH and may develop a potential molecular target for the treatment of ONFH.

## Figures and Tables

**Table 1:** Primers and probes for the target SNP genotypes of SOX9 following LDR.

dbSNP	Primer^a^	Probe^b^	Product size^c^
rs12601701 [A/G]	Sense: TCATACAACTAAGTACAGACG	TA:TTTTTTGACACAGAAATAGGTCCACACTACA	61/A,
	Antisense: TGCATACAATAGCTTCGTATC	TG:TTTTTTTTTGACACAGAAATAGGTCCACACTACG TR:-P-CGGACTTTTTTCTCCTAGGAAAGGATTTTT-FAM	64/G

rs1042667 [A/C]	Sense: CACTGGGAACAACCCGTCTAC	TA:TTTTTTTTTTGAGGAGGCCTCCCACGAAGGGCGAA	68/A,
	Antisense: TCCAAAGGGAATTCTGGTTGG	TC:TTTTTTTTTTTTTGAGGAGGCCTCCCACGAAGGGCGAC TR:-P-GATGGCCGAGATGATCCTAAAAATAACCGAAGA-FAM	71/C

^a^Primer to amplify the gene fragment of the target dbSNP site.

^b^Probe to discriminate the target SNP genotype.

^c^Product size indicates the size of the ligase reaction product from the different SNP genotypes.

**Table 2:** Association of SOX9 gene polymorphism with ONFH risk.

dbSNP ID	Position	Group	Genotype (*n*)			MAF	HWE^a^	Codominants (11 versus 12 versus 22)	Dominants 12 + 22 versus 11	Recessive 22 versus 11 + 12	Allele 2 versus 1
			11	12	22			OR (95% CI)	OR (95% CI)	OR (95% CI)	OR (95% CI)
								*P* ^c^	*P* ^c^	*P* ^c^	*P* ^c^
rs1042667 (A>C)	3′UTR^b^		CC	AC	AA						
		Control	46	90	43	0.491	0.835	1.244 (0.658–2.352)	0.971 (0.756–1.246)	0.930 (0.714–1.212)	0.906 (0.676–1.213)
		ONFH	51	92	39	0.467	0.937	0.502	0.815	0.930	0.508

rs12601701 (G>A)	Promoter		AA	GA	GG						
		Control	48	93	38	0.472	0.518	1.396 (0.737–2.644)	0.734 (0.452–1.194)	1.009 (0.582–1.749)	1.179 (0.879–1.581)
		ONFH	61	85	36	0.431	0.571	0.306	0.213	0.974	0.271

^a^ *P* values of deviation from Hardy-Weinberg equilibrium between the ONFH and control groups.

^b^3′untranslated region.

^c^Logistic regression analyses were used for calculations.

11: homozygotes for the major allele, 12: heterozygotes, and 22: homozygotes for the minor allele.

**Table 3:** Association of SOX9 gene polymorphisms with ONFH subgroups.

dbSNP	Subgroup	Genotype^*∗*^			MAF	Allele (2 versus 1)		Codominant (11 versus 12 versus 22)^*∗*^		Dominant (12 + 22 versus 11)		Recessive (22 versus 11 + 12)	
		11	12	22		OR (95% CI)	*P* ^#^	OR (95% CI)	*P* ^#^	OR (95% CI)	*P* ^#^	OR (95% CI)	*P* ^#^
rs1042667 (A>C)		AA	AC	CC									
	Ster^▲^	9	25	11	0.478	0.946	0.814	1.059	0.812	1.125	0.568	0.967	0.863
						(0.596~1.503)		(0.662~1.692)		(0.751~1.684)		(0.662~1.413)	
	Alc^▲^	19	35	14	0.463	1.198	0.370	0.861	0.694	0.964	0.903	0.894	0.535
						(0.807~1.780)		(0.408~1.817)		(0.135~1.736)		(0.627~1.274)	
	Idio^▲^	11	30	26	0.462	0.656	**0.040**	1.843	0.073	0.404	0.772	1.376	**0.047**
						(0.437~0.982)		(0.945~3.593)		(0.420~1.418)		(1.005~1.883)	

rs12601701 (G>A)		GG	GA	AA									
	Ster	9	23	13	0.456	1.069	0.779	1.053	0.896	1.014	0.968	1.038	0.939
						(0.672~1.699)		(0.481~2.283)		(0.519~1.980)		(0.721~1.496)	
	Alc	16	30	22	0.456	1.067	0.747	1.637	0.161	0.658	0.168	1.212	0.242
						(0.715~1.586)		(0.821~3.624)		(0.363~1.192)		(0.878~1.672)	
	Idio	11	26	30	0.358	1.410	0.095	1.790	0.085	0.757	0.370	1.343	0.065
						(0.941~2.113)		(0.922~3.476)		(0.412~1.391)		(0.982~1.835)	

^▲^Alc: Alcohol-induced; Ster: steroid- induced; Idio: idiopathic; ^*∗*^11: homozygotes for the major allele; 12: heterozygotes; and 22: homozygotes for the minor allele.

^#^Logistic regression analyses were used. Bold: *P* value < 0.05.

**Table 4:** Haplotype frequencies of the SOX9 gene between ONFH and control groups.

	Haplotype		Frequency		OR (95% CI)	*P* ^#^
	rs1042667	rs12601701	Controls *n* (%)	Patients *n* (%)		
Hap1	A	A	10.09 (2.8)	21.49 (5.9)	2.199 (1.026–4.717)	**0.038**
Hap2	A	G	165.91 (46.3)	148.51 (40.8)	0.817 (0.608–1.099)	0.182
Hap3	C	A	178.91 (50.0)	185.51 (51.0)	1.073 (0.800–1.441)	0.636
Hap4	C	G	3.09 (0.9)	8.49 (t2.3)	2.745 (0.740–10.179)	0.116

#: *χ* ^2^ test.

**Table 5:** Association of the rs1042667 and rs12601701 genotypes of SOX9 gene with the clinical phenotypes of ONFH.

dbSNP	Genotype	Sex *χ* ^2^ test		Disease course (months) ANOVA test	Etiological classification *n* (%) *χ* ^2^ test			Unilateral or bilateral hips lesions *n* (%) *χ* ^2^ test		Clinical stage *n* (%) *χ* ^2^ test		
		Men *n* (%)	Women *n* (%)		Alc	Ster	Idio	Unilateral	Bilateral	Stage II	Stage III	Stage IV
rs1042667	AA	30 (23.8)	9 (16.1)	86.72 + 74.80	19 (27.9)	9 (20.0)	11 (16.4)	14 (17.7)	25 (24.5)	2 (15.4)	10 (19.2)	26 (23.2)
	AC	63 (50.0)	29 (51.8)	66.66 + 72.85	35 (51.5)	25 (55.6)	30 (44.8)	39 (49.4)	52 (51.0)	8 (61.5)	30 (57.7)	52 (46.4)
	CC	33 (26.2)	18 (32.1)	71.88 + 62.21	14 (20.6)	11 (24.4)	26 (38.8)	26 (32.9)	25 (24.5)	3 (23.1)	12 (23.1)	34 (30.4)
	*P*	0.450		0.332	0.131			0.352		0.646		

rs12601701	AA	41 (32.6)	20 (35.7)	65.13 + 60.99	22 (32.4)	13 (28.9)	26 (38.8)	36 (45.6)	25 (24.5)	3 (23.1)	20 (38.5)	36 (32.2)
	AG	59 (46.8)	26 (46.4)	72.42 + 73.97	30 (44.1)	23 (51.1)	30 (44.8)	32 (40.5)	52 (51.0)	7 (53.8)	25 (48.0)	51 (45.5)
	GG	26 (20.6)	10 (17.9)	84.94 + 77.19	16 (23.5)	9 (20.0)	11 (16.4)	11 (13.9)	25 (24.5)	3 (23.1)	7 (13.5)	25 (22.3)
	*P*	0.874		0.411	0.729			**0.009**		0.636		

**Table 6:** Haplotypes association of the SOX9 gene with hips lesions and clinical stages of ONFH.

Clinical phenotypes	Subtype	rs1042667(A), rs12601701(A)			rs1042667(A), rs12601701(G)			rs1042667(C), rs12601701(A)			rs1042667(C), rs12601701(G)		
		*n* (%)	*P*	OR (95% CI)	*n* (%)	*P*	OR (95% CI)	*n* (%)	*P*	OR (95% CI)	*n* (%)	*P*	OR (95% CI)
Hip lesions	Unilateral	14.09 (8.9)	**0.032**	0.377 (0.150–0.947)	52.91 (33.5)	**0.013**	1.722 (1.120–2.647)	89.91 (56.9)	**0.048**	0.657 (0.432–0.997)	1.09 (0.7)	0.073	5.317 (0.703–40.228)
	Bilateral	7.24 (3.6)			94.73 (46.4)			94.73 (46.4)			7.27 (3.6)		

Clinical stages	Stage IV	10.30 (4.6)	**0.041**	0.408 (0.408–0.987)	93.70 (41.8)	0.457	1.198 (0.743–1.932)	112.70 (50.3)	0.786	0.937 (0.589–1.493)	7.30 (3.3)	0.063	—
	Stage III	11.00 (10.6)			39.00 (37.5)			54.00 (51.9)			0.00 (0)		
